# A Comprehensive Review on Advancements in Wearable Technologies: Revolutionizing Cardiovascular Medicine

**DOI:** 10.7759/cureus.61312

**Published:** 2024-05-29

**Authors:** Vaishnavi Bhaltadak, Babaji Ghewade, Seema Yelne

**Affiliations:** 1 Respiratory Medicine, School of Allied Health Science, Datta Meghe Institute of Higher Education and Research, Wardha, IND; 2 Respiratory Medicine, Jawaharlal Nehru Medical College, Datta Meghe Institute of Higher Education and Research, Wardha, IND; 3 Nursing, Shalinitai Meghe College of Nursing, Datta Meghe Institute of Higher Education and Research, Wardha, IND

**Keywords:** patient-centered care, personalized health tracking, artificial intelligence, remote patient monitoring, cardiovascular medicine, wearable technologies

## Abstract

Wearable technologies have emerged as powerful tools in healthcare, offering continuous monitoring and personalized insights outside traditional clinical settings. These devices have garnered significant attention in cardiovascular medicine for their potential to transform patient care and improve outcomes. This comprehensive review provides an overview of wearable technologies' evolution, advancements, and applications in cardiovascular medicine. We examine the miniaturization of sensors, integration of artificial intelligence (AI), and proliferation of remote patient monitoring solutions. Key findings include the role of wearables in the early detection of cardiovascular conditions, personalized health tracking, and remote patient management. Challenges such as data privacy concerns and regulatory hurdles are also addressed. The adoption of wearable technologies holds promise for shifting healthcare from reactive to proactive, enabling precision diagnostics, treatment optimization, and preventive strategies. Collaboration among healthcare stakeholders is essential to harnessing the full potential of wearables in cardiovascular medicine and ushering in a new era of personalized, proactive healthcare.

## Introduction and background

Wearable technologies encompass devices worn or attached to the body to monitor physiological parameters, track activity levels, and provide real-time health insights [1]. These devices often incorporate sensors, data processing capabilities, and wireless connectivity, enabling continuous monitoring and analysis of user data. Their significance lies in their potential to revolutionize healthcare by enabling personalized, proactive, and continuous monitoring outside traditional clinical settings [2].

In recent years, wearable technologies have garnered significant attention for their potential to transform cardiovascular medicine. These devices offer noninvasive and convenient methods for monitoring vital signs, detecting anomalies, and managing chronic conditions such as hypertension, arrhythmias, and heart failure [3]. By providing continuous data streams and actionable insights, wearables empower individuals to take proactive measures to improve their cardiovascular health and facilitate early intervention when necessary [4].

This review aims to provide a comprehensive overview of the advancements in wearable technologies and their role in revolutionizing cardiovascular medicine. By synthesizing existing literature and highlighting key developments, this review aims to elucidate the field's current state, identify emerging trends, and discuss the potential implications for clinical practice and research. Additionally, this review will address the challenges and limitations associated with wearable technologies in cardiovascular medicine and explore future directions for innovation and integration into healthcare systems.

## Review

Evolution of wearable technologies

Historical Background

The history of wearable technology unfolds as a rich tapestry of innovation and evolution spanning centuries. Returning to the earliest recorded mention of eyeglasses in 1268 and culminating in aviator Alberto Santos-Dumont's creation of the first wristwatch in 1907, wearables have continually adapted to meet evolving needs and technological advancements [5]. In the 1960s, Edward Thorp and Claude Shannon pioneered the development of the first wearable computer, initially utilized for predictive purposes in games such as roulette [6]. This period also witnessed the emergence of innovations like the head-mounted stereophonic television display, patented by Heilig in 1960 [5]. The 1980s were marked by a burgeoning desire, inspired by movies like "The Terminator," to materialize real-life computer displays for the eyes, hinting at the future development of devices like Google Glass [6]. Meanwhile, the 1990s saw wearables momentarily recede in prominence amid the rapid evolution of mobile phones. However, concepts such as computerized clothing and body-mounted cameras were explored, as evidenced by workshops like "Wearables in 2005" hosted by the Defense Advanced Research Projects Agency (DARPA) [5].

The dawn of the 2000s witnessed a notable uptick in wearable device production, with the introduction of Bluetooth headsets, digital pacemakers, and collaborative ventures such as those between Nike and Apple to develop fitness tracking devices [6]. The 2010s were heralded as "The Year of the Wearable," characterized by groundbreaking advancements like the Apple Watch and Google Glass and the surge of health and fitness wearables such as Fitbit and Garmin, which came to dominate the market [7]. This era also saw the advent of smart glasses like Google Glass, laying the groundwork for the emergence of virtual reality and augmented reality devices like Oculus Rift and Microsoft HoloLens [7].

Milestones in Development

The release of the Hamilton Pulsar in 1972 marked a pivotal moment in wearable technology, introducing the world to the first digital watch and heralding the fusion of technology with fashion [8]. In 1999, the advent of Bluetooth headsets revolutionized communication by enabling wireless connectivity to mobile phones. This innovation significantly enhanced hands-free communication on the go, offering newfound convenience and mobility [8]. The launch of Fitbit in 2008 represented a milestone in fitness tracking. This innovative device allowed users to monitor physical activity, heart rate, and sleep patterns, empowering individuals to adopt healthier lifestyles through informed decision-making [8]. Despite its brief consumer presence, the introduction of Google Glass in 2013 left a lasting impact on wearable technology. These augmented reality glasses sparked interest in smart eyewear with diverse applications across industries, hinting at the future potential of wearable computing [8].

The debut of the Apple Watch in 2015 set a new standard for smartwatches, seamlessly integrating with iPhones and offering a myriad of features ranging from health monitoring to mobile payments. Its sleek design and extensive functionalities quickly became a coveted accessory [8]. In 2016, devices like the Oculus Rift brought virtual reality experiences into the mainstream, transforming entertainment and educational landscapes. These immersive headsets revolutionized how users interacted with digital content, opening up new possibilities for interactive storytelling and simulation [8]. The healthcare sector witnessed a significant transformation in 2017 with the emergence of electrocardiogram (ECG)-capable Apple Watches and glucose-monitoring wearables. These devices empowered individuals to monitor their health more effectively, offering valuable insights and facilitating early intervention [8]. Smart clothing embedded with sensors gained momentum in 2019, allowing users to monitor vital signs, track physical performance, and optimize sports training regimens. This innovation represented a convergence of fashion and technology, promising enhanced comfort and performance for wearers [8].

**Figure 1:**
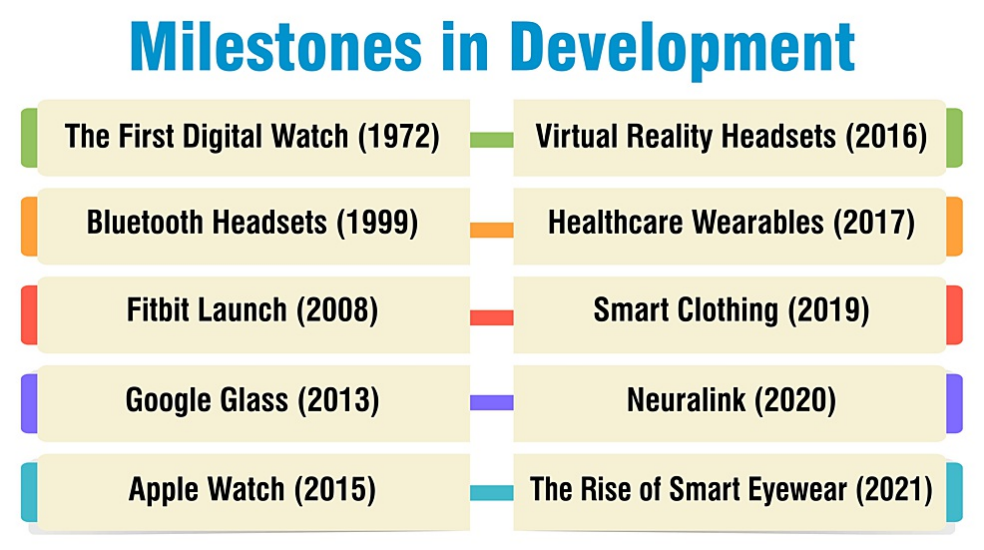
Milestones in development The image is created by the corresponding author

Elon Musk's Neuralink venture in 2020 delved into the realm of brain-machine interfaces, offering the potential for direct device control with thoughts. This groundbreaking technology held promise for revolutionizing human-computer interaction and assisting individuals with disabilities [8]. In 2021, the rise of stylish smart eyewear like Amazon Echo Frames and Facebook's Ray-Ban Stories blurred the lines between fashion and function. These wearable devices offered features such as calls, photos, and voice-assisted artificial intelligence (AI), further integrating technology into everyday life [8]. Figure 1 shows milestones in development.

Current State of the Art

The contemporary landscape of wearable technology is marked by a wide array of devices seamlessly woven into our daily routines, offering functionalities extending beyond conventional health monitoring. These wearables have evolved into smart jewelry, smart clothing, wearable cameras, fitness trackers, smart glasses, and even implantable health-monitoring pills [9]. They have transitioned from mere health aids to indispensable tools facilitating communication, productivity, and entertainment. Recent research underscores the pivotal role of wearables across diverse sectors, including healthcare, telemedicine, lifestyle, gaming, sports, fitness, environment, safety, and prevention [10]. No longer confined to basic health tracking, wearable technology has expanded its purview to encompass prosthetics, exoskeletons, soft robotics, and other state-of-the-art innovations leveraging AI, machine learning (ML), and deep learning for postprocessing and real-time data analysis [10]. This progression highlights wearables' increasing sophistication and adaptability in addressing various industries' needs and challenges. Furthermore, wearables are steadily infiltrating the workplace to augment connectivity, communication, and productivity. They offer tailored solutions to address the demands of flexible work arrangements. They are envisioned to streamline tasks such as booking meeting rooms, checking calendars, and facilitating on-the-go work activities [9]. The potential of wearables to enhance workplace efficiency is palpable, with many respondents acknowledging their positive impact on interconnected living and work environments [9].

Types of wearable technologies in cardiovascular medicine

Smartwatches and Fitness Trackers

Smartwatches and fitness trackers are ubiquitous examples of wearable devices employed in cardiovascular medicine. Armed with an array of sensors, these gadgets can meticulously track heart rate, activity levels, and even blood pressure, furnishing invaluable data for health maintenance [11-13]. Embraced by consumers worldwide, smartwatches and fitness trackers have soared in popularity, with approximately 20% of US residents owning a smart wearable device, and the global market is poised to soar to US$70 billion by 2025 [11]. Their significance lies in their pivotal role in remote monitoring, dispensing personalized health insights, and facilitating disease management, offering a more holistic perspective on health compared to conventional sporadic assessments [12]. Crafted to gauge physical activity, heart rate, heart rhythm, and various physiological parameters, these wearable devices prove instrumental in cardiovascular risk assessment, preemptive health strategies, diagnostic endeavors, and ongoing condition management [12,13]. Despite their potential merits, several impediments loom, including concerns regarding device accuracy, clinical validity, regulatory protocols, privacy safeguards, and the imperative for large-scale trials [11,13,14]. A concerted, collaborative endeavor among stakeholders emerges as imperative to surmount these challenges and seamlessly integrate wearable technologies into the clinical continuum, thereby optimizing patient care [14]. Through such collective efforts, the transformative potential of wearable technologies in cardiovascular medicine can be fully harnessed, paving the way for enhanced patient outcomes and more efficient healthcare delivery.

ECG Monitors

ECG monitors, also called cardiac event monitors, are indispensable wearable devices in diagnosing heart conditions that may not manifest daily. These compact, portable devices are adept at recording heart rate and rhythm over prolonged periods, providing clinicians with valuable insights into irregularities that might evade detection during routine medical evaluations [15]. Distinguished by their convenience and continuous monitoring capabilities, ECG monitors offer a practical alternative to conventional ECGs, ensuring uninterrupted data collection in diverse settings [15]. Patients wear these monitors for several weeks to capture sporadic abnormal heart rhythms that may elude detection during fleeting medical consultations. In various configurations, cardiac event recorders necessitate constant wear or carriage by the individual to ensure seamless monitoring throughout the day [15]. Functionally, ECG monitors operate by employing sensors affixed to the chest to capture heart rhythms, subsequently wirelessly transmitting this data to the monitor for recording. The collected information can then be transmitted to healthcare providers via phones or computers for comprehensive analysis and diagnosis. Depending on the specific monitor type, individuals may be required to affix sensors to their chest, wear the monitor on their wrist, or hold it against their chest [15]. Some monitors necessitate manual activation upon symptom onset, while others are designed to automatically initiate recording upon detecting abnormal heart rhythms [15]. In essence, ECG monitors play a pivotal role in cardiovascular care by facilitating continuous, noninvasive monitoring of heart function, thereby aiding in the timely diagnosis and management of cardiac issues. Their versatility and convenience make them indispensable tools in the arsenal of modern healthcare professionals striving to ensure optimal patient outcomes.

Blood Pressure Monitors

Wearable technologies have emerged as indispensable assets in cardiovascular medicine, particularly in managing health conditions such as hypertension. Among these innovations, blood pressure monitors are pivotal in facilitating the accurate diagnosis and ongoing monitoring of hypertension-related parameters [16]. Offering the distinct advantage of frequent blood pressure measurements with minimal disruption to patients' daily routines, these devices are instrumental in identifying nuanced conditions like masked hypertension and pathological blood pressure variability [16].

The continual advancements in wearable blood pressure monitoring devices hold tremendous promise in enhancing cardiovascular prognosis. By enabling early diagnosis, facilitating appropriate treatment interventions, and bolstering patient adherence to prescribed regimens, these technologies wield significant potential to revolutionize patient care [16]. Numerous studies have scrutinized the accuracy and reliability of wearable blood pressure monitors, juxtaposing their performance against traditional oscillometry-based devices. For instance, smartwatches outfitted with photoplethysmography technology have demonstrated comparable accuracy levels to standard devices, rendering them suitable for adult blood pressure measurement [16].

Moreover, integrating novel features in blood pressure variability monitoring represents a watershed moment in cardiovascular medicine. This augmentation facilitates a more nuanced interpretation of blood pressure data in real-life scenarios, thereby furnishing healthcare practitioners with invaluable insights to devise tailored management strategies for hypertension [17]. In essence, wearable blood pressure monitoring devices epitomize a significant stride forward in cardiovascular medicine, proffering a pragmatic and streamlined approach to continuous blood pressure level surveillance. With their potential to refine diagnosis, optimize management, and ultimately enhance patient outcomes, these devices underscore the imperative of integrating wearable technologies into the clinical landscape to better cardiovascular healthcare delivery [17].

Wearable Biosensors

Wearable biosensors play a pivotal role in cardiovascular medicine, furnishing continuous and noninvasive monitoring of many physiological parameters. These biosensors, seamlessly integrated into wearable devices such as smartwatches and clothing, deliver real-time data concerning heart rate, blood pressure, ECG, and various other biophysical and biochemical indicators [18,19]. By enabling remote patient tracking, facilitating early diagnosis, and supporting personalized medicine, these biosensors significantly bolster the management of cardiovascular diseases (CVDs) on both individual and population scales [19].

The utilization of wearable biosensors in cardiovascular medicine is rapidly advancing, propelled by groundbreaking strides in biosensing technologies and the incorporation of smart materials, AI, and big data analytics [18,19]. Research endeavors underscore the immense potential of wearable biosensors in enhancing disease screening, monitoring, and delivering personalized care within cardiovascular medicine. Seamlessly integrated into diverse wearable platforms such as wristbands, patches, and contact lenses, these biosensors offer many applications beyond conventional monitoring devices [18,19].

Nevertheless, formidable challenges persist, including accuracy, stability, energy harvesting, and system integration concerns. Addressing these challenges necessitates further technical innovations to fortify the reliability and efficacy of wearable biosensors within healthcare settings [18,19]. Undoubtedly, wearable biosensors represent an exciting frontier in cardiovascular medicine, furnishing invaluable insights into patients' health status while facilitating early detection and precise disease prediction. Furthermore, the fusion of ML techniques with wearable devices amplifies their potential to promote well-being and enhance patient outcomes within cardiovascular care [18,19].

Smart Clothing

The utilization of wearable technologies in cardiovascular medicine, including smart clothing embedded with sensors, is swiftly gaining traction as a valuable resource for monitoring and managing cardiovascular health. These innovative devices furnish continuous data on many physiological parameters, encompassing heart rate, activity levels, and blood pressure, thus affording a more holistic perspective on health status than conventional sporadic assessments [14,20]. Demonstrating promise in disease screening and monitoring, wearable devices hold the potential to tailor and optimize the management of CVDs on both individual and population-wide scales [14,20]. Despite their enticing benefits, hurdles persist, ranging from concerns regarding device accuracy and clinical validation to regulatory policies, privacy considerations, and the imperative for large-scale trials [14,21]. These challenges impede the widespread adoption of wearable technologies in clinical practice, necessitating collaborative efforts among stakeholders to surmount them effectively [14]. Through concerted collaboration, stakeholders can navigate these obstacles and ensure the equitable integration of wearable technologies into cardiovascular medicine, thereby realizing their full potential in enhancing patient care and outcomes.

Advancements in wearable technologies

Miniaturization and Portability

The paradigm of miniaturization and portability within wearable technologies has profoundly reshaped the healthcare and consumer electronics landscapes. This trend has spurred the development of smaller, more efficient devices, enhancing their portability and user convenience. Notably, the evolution of medical devices such as pacemakers, glucose monitoring systems, and hearing aids exemplifies how miniaturization has transformed bulky, traditional devices into compact, implantable, or wearable solutions [22,23]. The ramifications of miniaturizing medical devices extend beyond mere comfort improvements, fostering less invasive procedures, and enabling real-time monitoring capabilities. Pioneering devices like Medtronic's Micra, recognized as the world's smallest pacemaker, and Dexcom's G6 continuous glucose monitoring (CGM) system underscore the profound impact of miniaturization on healthcare, offering patients personalized and efficient solutions [22]. Furthermore, the integration of AI and ML in devices such as the Eversense CGM System amplifies their capabilities, furnishing real-time insights and enhancing disease management [22].

In consumer electronics, the miniaturization trajectory has witnessed devices evolve from standalone entities to fully integrated components of smartphones, rendering technology more accessible and convenient for users [23]. Wearable devices, including smartwatches, fitness trackers, and even smart fabrics, have surged in popularity due to their compact dimensions, real-time monitoring functionalities, and ability to track diverse health parameters [22,23]. The miniaturization trajectory is poised to delve into nanomedicine, nanobots, and nanoscale biosensors, promising even more groundbreaking advancements in diagnosis, drug delivery, and disease monitoring [22]. As miniaturization continues to drive innovation across sectors, its transformative potential in enhancing healthcare delivery and consumer electronics remains a compelling narrative shaping the future of technology.

Enhanced Sensor Accuracy and Reliability

Optimal sensor placement on the body is a critical determinant of accuracy in wearable technology. The efficacy of sensor placement varies depending on the physiological parameter being measured, with different locations offering superior accuracy due to their proximity to relevant body features such as the heart or blood vessels [24]. Calibration ensures accuracy by aligning sensor output with a reference standard or known value. This calibration process mitigates errors from factors like sensor drift and environmental changes. Calibration can be executed manually or automatically before or after data collection, enhancing accuracy and precision [24,25].

Validation is crucial in assessing sensor output's accuracy, precision, sensitivity, specificity, and repeatability. By comparing sensor data against gold standards or criterion measures, validation facilitates the identification of errors and uncertainties that may compromise data quality and reliability [24,26]. Integrating outputs from multiple sensors through hardware or software mechanisms bolsters accuracy by mitigating individual sensor limitations such as noise, drift, and placement errors. This integrated approach enhances accuracy and improves functionality and usability by enabling comprehensive data analysis and feedback [25]. Implementing design strategies focused on sensor accuracy and reliability is paramount for ensuring high-quality data collection. Factors such as signal conditioning, amplification, power provision, and multipoint calibration play pivotal roles in optimizing sensor performance and enhancing the overall efficacy of wearable technology [25].

Integration of AI and ML

The infusion of AI and ML into wearable technologies has markedly augmented their capabilities, particularly in healthcare. By amalgamating AI with wearable sensors, these devices can discern patterns, analyze data more efficiently, and furnish personalized insights for users. ML algorithms embedded within wearables facilitate real-time monitoring of vital signs, activity levels, and emotional states, enhancing health management and diagnostics [27,28]. The advent of AI-driven wearable sensors has revolutionized the precision and efficacy of data collection and processing, affording error correction mechanisms and facilitating enhanced data analysis. These technologies are pivotal in disease prognosis, diagnosis, treatment feedback, and personalized health monitoring [28]. Leveraging AI techniques such as ML and deep learning within wearable devices has ushered in a plethora of new applications, including fall detection, gait analysis, sleep monitoring, mental health tracking, and beyond [28].

Improved Connectivity and Data Management

Enhanced connectivity and streamlined data management are pivotal drivers propelling wearable technologies' evolution, particularly within healthcare. These devices are progressively advancing in their ability to track health metrics, monitor vital signs, and furnish real-time health alerts to users [29]. By collecting and analyzing long-term continuous data on physiological functions, wearable devices offer clinicians a more comprehensive understanding of patient's health status than conventional sporadic assessments [12]. The integration of Bluetooth Low Energy (BLE) communication technology further elevates the capabilities of wearables in healthcare applications, enabling the assessment of nerve conduction, muscle contractions, and muscle response in injured tissue [30]. Moreover, strides in communication networks, exemplified by fifth-generation (5G) technology, bolster broadband networks to accommodate demanding requisites such as low latency and high data rates, facilitating seamless data transmission between wearables and remote systems [30]. Wearable devices can intercommunicate, utilizing various transmission mediums like BLE, Zigbee, or Wi-Fi, enabling efficient data transfer and analysis for personalized health insights [30]. Integrating wearable device data directly with electronic health records or practice management software amplifies the accessibility and usability of collected data for healthcare professionals, streamlining the process of informed decision-making and patient care [31].

Applications in cardiovascular medicine

Remote Patient Monitoring

Remote patient monitoring, facilitated by wearable devices, is revolutionizing healthcare by empowering individuals to take an active role in their healthcare journey, fostering accountability, and delivering valuable real-time data to patients and healthcare providers [32]. These devices, from glucose monitors to heart rate monitors and fitness trackers, enable continuous health monitoring beyond traditional clinical settings, facilitating early interventions and personalized care [32,33]. Integrating smartphones with wearables further amplifies remote patient monitoring capabilities, facilitating seamless data collection, analysis, and immediate feedback. Leveraging smartphones with sensors and the Internet of Things (IoT) connectivity bridges the gap between conventional health monitoring and digital medicine, offering cost-effective solutions for patients and healthcare providers [33]. By harnessing smartphones for real-time health metrics and remote monitoring, healthcare professionals can identify subtle changes early on, enabling prompt medical intervention and mitigating the risks of unexpected health events [33]. Patient adherence to wearable technologies stands as a pivotal factor for the success of remote patient monitoring initiatives. Adherence is influenced by various factors, including the complexity of technology, compatibility with lifestyle, physical constraints, forgetfulness, lack of motivation, age-related challenges, and disease-specific considerations [34]. Overcoming these challenges necessitates innovative solutions and user-centric design approaches to improve patient adherence and enhance healthcare outcomes through remote patient monitoring [34].

Early Detection of Cardiovascular Conditions

The early detection of cardiovascular conditions is undergoing a significant transformation with the integration of AI technology alongside ECGs. Researchers and clinicians at the Mayo Clinic are harnessing AI to detect earlier heart diseases, focusing on conditions such as amyloidosis, aortic stenosis, low ejection fraction, and hypertrophic cardiomyopathy (HCM) [35]. This innovative approach enables the prediction of a patient's likelihood of developing these conditions, facilitating proactive management and potentially enhancing patient outcomes. Moreover, AI-enabled ECG algorithms play a pivotal role in the early detection of conditions like low ejection fraction, which, if left untreated, can lead to heart failure. By employing AI to scrutinize ECG data, clinicians can discern subtle patterns indicative of conditions like HCM that may evade detection in traditional tests, thereby enabling early intervention and treatment [35].

Personalized Health Tracking and Management

Personalized health tracking and management through wearable technologies represent a promising avenue for enhancing healthcare in cardiovascular medicine. These devices continuously monitor physiological parameters such as heart rate, activity levels, and blood pressure, thereby furnishing personalized insights into an individual's health status [11,14]. By harnessing wearable devices, clinicians can access invaluable data to predict, prevent, diagnose, and treat CVDs more effectively, ultimately leading to improved outcomes at both individual and population levels [14]. Additionally, wearable technologies facilitate personalized feedback and guidance, exemplified by systems like personal activity intelligence (PAI), which tailors activity targets based on individual data rather than generic standards [11]. These devices empower individuals to monitor their health proactively and enable healthcare professionals to make more informed decisions by integrating wearable data into clinical workflows to optimize patient care [12,36]. Despite challenges such as device accuracy, clinical validity, and privacy concerns, collaborative efforts among stakeholders are indispensable for navigating these obstacles and ensuring the equitable adoption of wearable technologies in cardiovascular medicine [20,36].

Rehabilitation and Preventive Care

Wearable technologies are pivotal in rehabilitation and preventive care, offering innovative solutions to monitor and improve patient outcomes. These devices, from sensors integrated into clothing to smart wearable gadgets, furnish valuable data on body movements, physiological parameters, and vital signs, thus facilitating personalized rehabilitation monitoring and sports performance tracking [37-39]. Particularly in postsurgical rehabilitation, wearable sensors enable remote monitoring of patient's progress and activities, potentially leading to more effective managed care and enhanced recovery outcomes [39]. In stroke rehabilitation, wearable technology presents a promising avenue for refining the diagnosis and treatment of upper-limb motor impairment. These devices facilitate objective and quantifiable data collection on patients' capabilities, augmenting the subjective assessments conducted by healthcare specialists and furnishing a more precise understanding of motor function during daily life activities [38]. By streamlining evaluation times, providing continuous monitoring, and delivering personalized insights, wearable sensors contribute to better-targeted care and individualized therapies for stroke patients [38]. Overall, wearable rehabilitation and preventive care technologies are evolving swiftly, proffering solutions to elevate patient monitoring, bolster rehabilitation outcomes, and enable remote tracking of health parameters. Despite device accuracy, regulatory policies, and privacy concerns, collaborative efforts among stakeholders are imperative to surmount these obstacles and seamlessly integrate wearables into clinical workflows for optimized patient care [11,40].

Challenges and limitations

Data Privacy and Security Concerns

Data privacy and security concerns loom large in wearable devices within cardiovascular medicine. These devices, which gather personal health data such as heart rate, activity levels, and sleep patterns, prompt ethical dilemmas regarding data protection and privacy [41]. Given the sensitive nature of this data, often stored in the cloud and shared with third-party apps and services, privacy breaches and misuse are pronounced if proper safeguards are not in place [41]. Unauthorized access or misuse of personal health data raises serious ethical and privacy concerns, underscoring the critical need for robust data security measures to safeguard individuals' information [41]. More robust regulations and industry self-regulation are imperative to tackle these concerns to ensure the appropriate use and protection of personal health data collected by wearable devices [41]. While existing laws like the General Data Protection Regulation (GDPR) and the Health Insurance Portability and Accountability Act (HIPAA) offer some level of protection, there is a pressing need for more comprehensive regulatory frameworks to safeguard personal health information effectively [41]. Moreover, prioritizing informed consent and user autonomy in data collection and use is paramount to mitigate the risks of privacy breaches and data misuse, which can have far-reaching repercussions for individuals and society [41].

Regulatory hurdles

Navigating the regulatory landscape surrounding wearable devices in cardiovascular medicine entails addressing evolving requirements for market access, authorization/certification, reimbursement, data protection, and data security [11,42]. These regulatory hurdles can pose significant barriers to the widespread adoption of smart wearables for cardiac monitoring despite the rapid progress in medical products and technologies within this domain [42]. Regulatory and legal frameworks governing market access are pivotal in ensuring the safety, efficacy, and privacy of wearable devices utilized in healthcare settings [42]. In the context of CVDs, securing market access for wearable devices involves traversing processes such as the European CE-marking and clearance or approval by the Food and Drug Administration (FDA) [42]. Manufacturers must also address considerations related to reimbursement and compensation, which vary across healthcare systems and can profoundly influence the utilization of these devices in clinical practice [42]. Furthermore, the legal landscape governing smart wearables is dynamic, with anticipated changes to accommodate the burgeoning market for wearables in cardiac monitoring and data collection for clinical predictive models [42]. To surmount these regulatory hurdles, stakeholders in the healthcare sector must collaborate to formulate comprehensive evaluation frameworks and pragmatic regulatory policies and conduct clinical trials to ascertain the benefits and safety of wearable devices in cardiovascular care [11,42]. By addressing these regulatory challenges early in product development, manufacturers can ensure compliance with evolving standards and facilitate the seamless integration of wearable technologies into routine clinical practice to optimize patient care [11,42].

Interoperability Issues

Interoperability challenges concerning wearable devices in cardiovascular medicine revolve around the fragmentation of medical device data, the necessity for a common language, protocols, and standards, and the difficulty of seamlessly integrating data from various devices [14,43]. Presently, medical device data are fragmented across different device types, encompassing consumer health applications, wearables, ambulance-mounted equipment, and movable and stationary devices in clinical settings [43]. This fragmentation underscores the imperative to establish common language, protocols, principles, and ground rules to facilitate smooth interfacing and data exchange among diverse devices. To tackle these interoperability challenges, concerted efforts are required to develop standards enabling data flow across various devices and applications. This ensures that interoperable networked devices can effectively furnish real-time, targeted alerts and streamline manual tasks [14,43]. Establishing a standards development organization featuring technical and systems expertise, industry participation, and stakeholder collaboration is pivotal in promoting interoperability in medical device technologies [43]. Through initiatives aimed at fostering research and development, enhancing clinical efficiency, and fortifying cybersecurity frameworks for data sharing, standards can catalyze the realization of the full potential of medical device interoperability, delivering benefits to patients and clinicians in cardiovascular medicine [43].

User Acceptance and Adherence

User acceptance and adherence to wearable devices in cardiovascular medicine are pivotal for their efficacy and integration into healthcare practices. Research reveals that among individuals with or at risk for CVD, fewer than one in four utilize wearable devices, with only half of those maintaining consistent daily usage [20]. Despite the potential of wearable devices to enhance cardiovascular health, current usage patterns may exacerbate disparities unless strategies are implemented to ensure equitable adoption [20]. Studies indicate that wearable devices are increasingly recognized as valuable tools for enhancing the detection and management of CVDs, particularly during health monitoring, including amid the COVID-19 pandemic [44]. Nonetheless, challenges to user acceptance, adoption, and participation persist as significant barriers to the widespread utilization of smart wearables for detecting and predicting CVDs [44]. Factors such as the complexity of navigating wearable device technology, limited understanding of the clinical role of devices, cost considerations, inconsistent internet access, data security apprehensions, and the absence of culturally diverse interventions can impact user acceptance and adherence to wearable devices in cardiovascular care [12]. Addressing these challenges requires a multifaceted approach encompassing user education, improved device usability, cost-effective solutions, robust data security measures, and culturally sensitive interventions tailored to diverse populations. By overcoming these barriers, healthcare providers and policymakers can foster greater acceptance and utilization of wearable devices, maximizing their potential to improve cardiovascular health outcomes.

Future directions

Emerging Technologies and Trends

**Figure 2:**
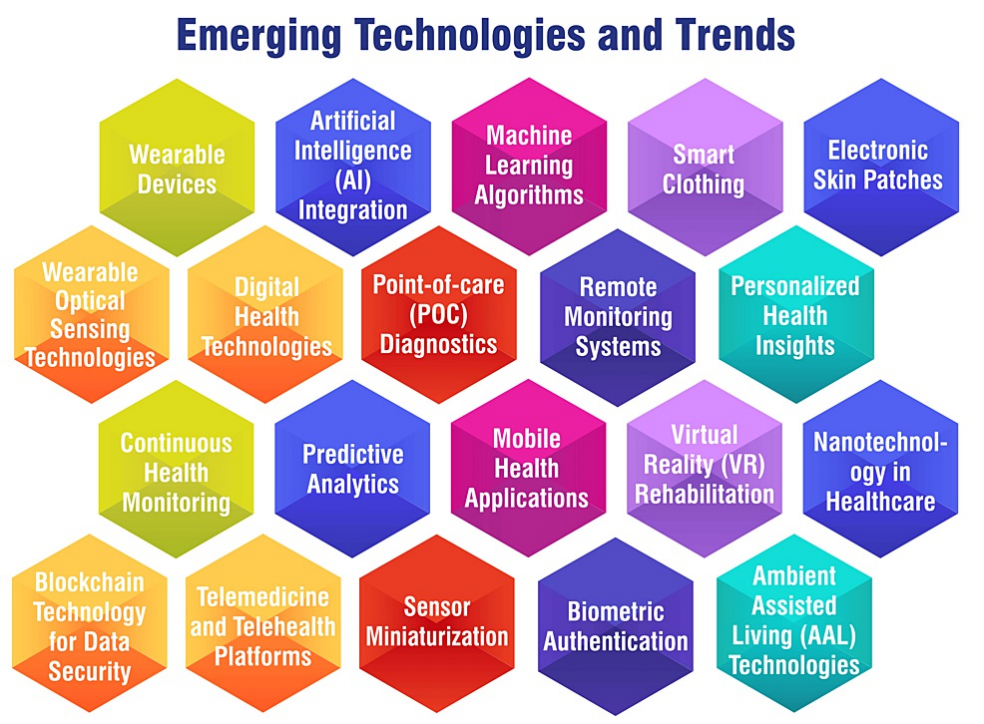
Emerging technologies and trends The image is created by the corresponding author

Emerging technologies and trends in cardiovascular medicine are reshaping the healthcare landscape, particularly through the proliferation of wearable devices and digital health technologies. These advancements present novel opportunities for remote monitoring, personalized health insights, and enhanced disease management among patients with cardiovascular conditions [11,45,46]. Wearable technologies, including smart clothing, electronic skin patches, and wearable optical sensing technologies, are broadening the scope of cardiovascular research and patient care [45]. These devices facilitate continuous monitoring of vital signs such as heart rate, activity levels, and blood pressure, furnishing healthcare professionals with invaluable data to inform treatment decisions and tailor interventions accordingly [11,45]. Furthermore, the integration of AI into cardiovascular imaging and in vitro diagnostics (IVD) at point of care (POC) is revolutionizing the detection and management of CVDs [45]. AI algorithms augment the accuracy and efficiency of diagnosing conditions like hypertension, thus guiding treatment strategies to improve patient outcomes [47]. Additionally, using ML in tandem with digital health technologies is paving the way for personalized risk assessments, real-time monitoring, and guideline-directed interventions in CVD prevention [46]. These innovative approaches hold immense promise in enhancing the efficacy, accessibility, and individualization of cardiovascular care, ultimately contributing to better patient outcomes and healthcare delivery. Figure 2 shows emerging technologies and trends.

Potential Impact on Clinical Practice

**Figure 3:**
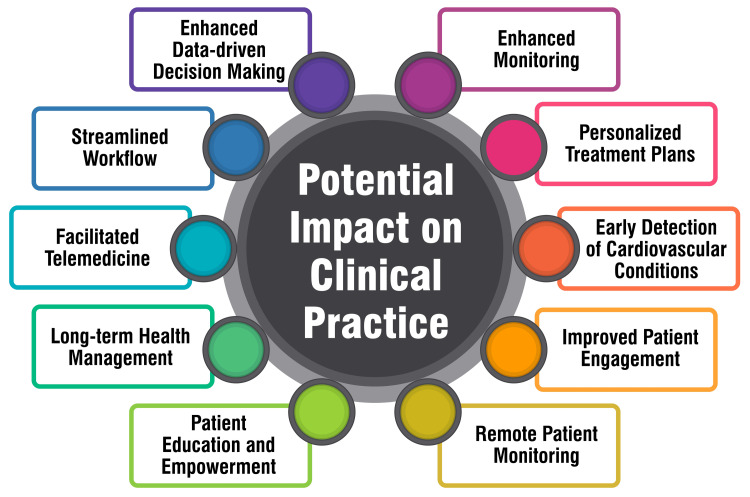
Potential impact on clinical practice The image is created by the corresponding author

The utilization of wearable devices among individuals with or at risk for CVD in the United States has exhibited promising trends while also underscoring disparities in adoption that could exacerbate existing health inequities. According to a cross-sectional study, 18% of individuals with established CVD and 26% at risk for CVD reported using wearable devices compared to 29% of the general population. Older age, lower educational attainment, and lower household income were associated with significantly diminished odds of wearable device use. Nonetheless, a notable proportion of users expressed readiness to share their health data with clinicians, suggesting a potential avenue for enhancing cardiovascular health outcomes through wearable technologies [20]. Despite these promising indications, the integration of wearable devices into clinical cardiology remains early, with the evidence base continually evolving. These devices hold the potential to personalize and enrich the management of CVDs on both individual and population-wide scales, ultimately culminating in improved outcomes. However, challenges such as device accuracy, clinical validity, regulatory policies, and privacy concerns necessitate attention to ensure the widespread adoption of wearable technologies in clinical practice. Collaborative endeavors among stakeholders are imperative to navigate these challenges effectively and facilitate the seamless integration of wearable devices into routine clinical workflows, optimizing patient care [11,14]. Figure 3 shows the potential impact on clinical practice.

Research Opportunities and Collaborations

**Figure 4:**
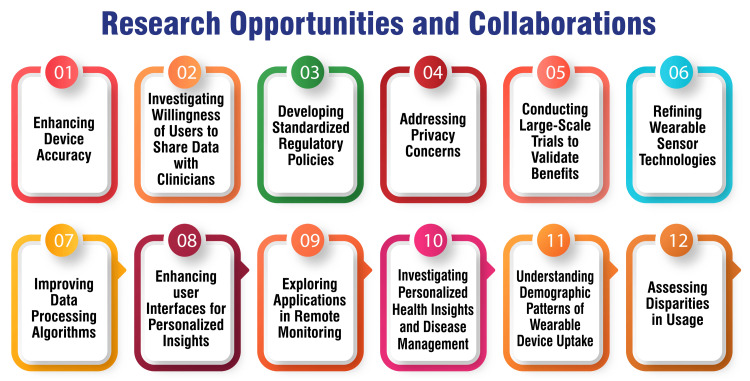
Research opportunities and collaborations The image is created by the corresponding author

Research opportunities and collaborations in wearable technologies for cardiovascular medicine are pivotal for advancing the integration of these devices into clinical practice. Critical areas for research encompass enhancing device accuracy, establishing clinical validity, developing standardized regulatory policies, addressing privacy concerns, and conducting large-scale trials to validate the benefits of wearable technologies [13,20,48]. Collaborative efforts among stakeholders, including healthcare providers, technology companies, policymakers, and patients, are imperative to surmount challenges and ensure the effective implementation of wearable devices in cardiovascular care [49]. Research opportunities include refining wearable sensor technologies, improving data processing algorithms, and enhancing user interfaces to furnish more personalized and actionable health insights [48]. Additionally, exploring the applications of wearable devices in remote monitoring, personalized health insights, and disease management can yield significant advancements in cardiovascular care [13,20,48]. Research initiatives should also focus on comprehending the demographic patterns of wearable device uptake, potential disparities in usage, and the willingness of users to share data with clinicians to enhance care [20]. Figure 4 shows research opportunities and collaborations.

## Conclusions

In conclusion, this review has provided a comprehensive overview of the advancements in wearable technologies and their transformative impact on cardiovascular medicine. By exploring the evolution, types, and applications of wearables, it is evident that these devices offer promising opportunities for improving patient care and outcomes. The miniaturization of sensors, integration of AI, and proliferation of remote monitoring solutions have enabled early detection of cardiovascular conditions, personalized health tracking, and remote patient management. However, challenges such as data privacy concerns and regulatory hurdles must be addressed to realize the potential of wearables in clinical practice fully. The widespread adoption of wearable technologies holds great promise for shifting healthcare toward a proactive and personalized approach, ultimately leading to improved patient outcomes and reduced healthcare costs. Collaboration among healthcare providers, researchers, policymakers, and industry stakeholders will be crucial in overcoming challenges and harnessing the full potential of wearables in cardiovascular medicine. By embracing innovation and prioritizing patient-centered care, we can pave the way for a future where wearable technologies play a central role in preventive, personalized, and proactive cardiovascular healthcare.
